# Genome Size Reversely Correlates With Host Plant Range in *Helicoverpa* Species

**DOI:** 10.3389/fphys.2019.00029

**Published:** 2019-01-30

**Authors:** Shen Zhang, Shaohua Gu, Xinzhi Ni, Xianchun Li

**Affiliations:** ^1^State Key Laboratory for Biology of Plant Diseases and Insect Pests, Institute of Plant Protection, Chinese Academy of Agricultural Sciences, Beijing, China; ^2^USDA-ARS Crop Genetics and Breeding Research Unit, Tifton, GA, United States; ^3^Department of Entomology and BIO5 Institute, University of Arizona, Tucson, AZ, United States

**Keywords:** C-value, endopolyploidy, evolution, host modification, host adaptation, *Helicoverpa* spp.

## Abstract

In organisms with very low percentages of transposable elements (TEs), genome size may positively or negatively correlate with host range, depending on whether host adaptation or host modification is the main route to host generalism. To test if this holds true for insect herbivores with greater percentages of TEs, we conducted flow cytometry to measure the endopolyploidy levels and C-values of the host modification (salivary gland and mandibular gland in head), host adaptation (midgut), and host use-independent tissues (male gonad, hemolymph, and Malpighian tubules) of the generalist *Helicoverpa armigera* and the head of its older specialist sister *H. assulta*. Larval salivary gland displayed a consecutive chain of endopolyploidy particles from 8Cx to higher than 32Cx and larval head and midgut had endopolyploidy nuclei clusters of 16Cx and 32Cx, whereas larval male gonad, hemolymph, and Malpighian tubules possessed no endopolyploidy nuclei of higher than 8Cx. The estimated genome size of the Solanaceae plant specialist *H. assulta* is 430 Mb, significantly larger than that of its older generalist sister *Heliothis virescens* (408 Mb) and those of its two generalist descendants *H. armigera* (394 Mb) and *H. zea* (363 Mb). These data not only reveal a negative correlation between host plant range and genome size in this terminal lineage, but also imply that *Helicoverpa* species appear to depend more on host modification than on host adaptation to achieve polyphagy.

## Introduction

Speciation often leads to expansion or contraction in genome size and host range (Moran, 2002; Parkhill et al., 2003; Cho et al., 2007; Thomson et al., 2008; Merhej et al., 2013; Langridge et al., 2015; Skoracka et al., 2018). Organisms may expand their host range by adapting to different host environments via evolving additional host use related metabolic pathways and genes involved in digestion and detoxification, or by modifying distinct host environments to one or a few standard nutrient environments to which they are specialized via secretion of effectors, toxins and host-cell interaction proteins (Parkhill et al., 2003; Thomson et al., 2008; Boothroyd, 2009; McNally et al., 2014; Langridge et al., 2015; Baroncelli et al., 2016). If the former—host adaptation—is the main route to host generalism, host range should be positively correlated with genome size. In contrast, host range is expected to be negatively correlated with genome size if the latter—host modification/standardization via secretions—is the main strategy to achieve host generalism (McNally et al., 2014).

Theoretical and empirical case studies supporting a positive or negative covariation between genome size and host range predicted by McNally et al. (2014) have been reported for pathogenic bacteria and fungi whose genomes are compact with a very low percentage of transposable elements (TEs) and non-coding intron and intergenic sequences. Theoretical modeling of genome size evolution for prokaryotes in stable and fluctuating environments projects that adaptation to a range of environmental conditions results in expansion in genome size (Bentkowski et al., 2015). Consistent with the host adaptation strategy, the genome sizes of three soil actinobacteria from the genus *Frankia*, which are facultative plant symbionts that form nitrogen-fixing root nodules upon infection of their host plants, range from 5.43 Mb for a narrow host range strain (*Frankia* sp. strain HFPCcI3) to 7.50 Mb for a medium host range strain (*Frankia alni* strain ACN14a) and to 9.04 Mb for a broad host range strain (*Frankia* sp. strain EAN1pec.) (Normand et al., 2007). The study on three plant pathogenic fungi also suggests that the transition from specialist to generalist *Fusarium* species is coupled with genome and protein family expansions (Ma et al., 2010). In a similar manner, genomic analysis of seven entomopathogenic fungus species within *Metarhizium* genus shows a parallel expansion of gene content and genome size from hemipteran specialists to transitional species (intermediate host range: coleopterans, lepidopterans) and then to generalists (more than seven orders of insect hosts) (Hu et al., 2014). Conversely, a phylogenetic comparative analysis of 191 human pathogenic bacteria species (121 zoonotic species, 70 azoonotic species) shows that the host range of these pathogens is positively correlated with their secretome size but negatively correlated with their genome size, which is consistent with the prediction of the host modification strategy (McNally et al., 2014).

The question is whether genome size and host range also covary in multicellular eukaryotes such as herbivorous insects, whose genomes have a greater percentage of repetitive TEs than those of pathogenic bacteria and fungi (Charlesworth and Barton, 2004). While genome size variation in pathogenic bacteria and fungi is driven largely by adaptation to different hosts and the related environments, both TE proliferations, the direct proximate driver, and adaptation to hosts and the related environments, the ultimate driver, play a role in shaping insect genome size (Alfsnes et al., 2017). TEs tend to proliferate and jump into new loci in the host genome, but the produced new copies may be purged out or retained, depending on the fitness consequence (deleterious, neutral or beneficial) of each proliferation/jumping event (Chen and Li, 2007; Hua-Van et al., 2011). For example, genome size expansion due to TE proliferation is subjected to developmental restriction in holometabolous insect orders (Gregory, 2002; Hanrahan and Johnston, 2011). Host plant expansion should require, as in the case of pathogenic bacteria and fungi, expansion of genes responsible for adapting to distinct host plants (e.g., allelochemical-metabolizing enzymes) (Li et al., 2007; Rane et al., 2016; Calla et al., 2017; Bansal and Michel, 2018) and/or modifying host plants (e.g., glucose oxidases and other plant defense-repressing genes) (Simon et al., 2015; Giron et al., 2016; Guiguet et al., 2016; Rivera-Vega et al., 2017; Yang et al., 2017; Basu et al., 2018) and proliferations of non-coding regulatory sequences responsible for regulation of host use related genes. Accordingly, we call insect tissues such as salivary gland and mandibular gland that express and secrete effector gene products (e.g., glucose oxidase, ATP hydrolyzing enzymes, calcium-binding proteins) into saliva or oral secretions to manipulate/modify host plant nutrients, structure and defense (Simon et al., 2015; Giron et al., 2016; Guiguet et al., 2016; Rivera-Vega et al., 2017; Yang et al., 2017; Basu et al., 2018) as host modification tissues. By the same criteria, we name insect tissues that produces genes product to sense/locate host plants (e.g., odorant receptors in antenna), digest plant tissues (digestion enzymes in midgut), and detoxify toxic plant defensive allelochemicals and protease inhibitors (detoxification enzymes in midgut and fat body) (Li et al., 2007; Simon et al., 2015; Rane et al., 2016; Calla et al., 2017; Bansal and Michel, 2018) as host adaptation tissues.

Transposable element proliferation and jumping events may lead to expansion of host use genes and their non-coding regulatory sequences since they are in close proximity to such loci (Chen and Li, 2007). If the expansion of host use related genes and non-coding regulatory sequences are too small to dictate the evolution of genome size relative to that of TEs, there will be lack of correlation between genome size and host plant range. This is likely to happen when species that belong to different higher taxonomic levels such as different families or even genera are compared. On the other hand, the contribution of host use related genes and regulatory sequences may be large enough to govern the evolution of genome size among closely related species or intraspecific populations that are similar in all life-history traits but differ mainly in host plant range. In the latter case, a positive or negative correlation may be found between genome size and host range.

Recently, Calatayud et al. (2016) found the genome sizes of three oligophagous stem borers (*Busseola nairobica, B. segeta, B. fusca*) are larger than those of two polyphagous (*Sesamia. calamistis, S. nonagrioides*) stem borers. The authors further examined the genome sizes and host plant ranges of 16 other lepidopteran species and found a positive covariance between their genome sizes and host ranges. Because the 5 stem borers belong to two different genera and the 16 lepidopteran species belong to 8 different families that may differ not only in host plant range but also in other life-history traits, it remains unclear whether this finding can be extrapolated to other insect herbivores.

In this study, we chose a group of *Helicoverpa* species (*H. armigera, H. zea* and *H. assulta*) to test if genome size covaries with host plant range. They were chosen because they are not only serious pests of economic importance, but also closely related to each other. *H. assulta* emerged earlier than did *H. armigera*, from which *H. zea* was established in the New World via a founder event around 1.5 million years ago (Mallet et al., 1993; Li et al., 2002a; Behere et al., 2007; Pearce et al., 2017). The three species are similar to each other in morphology and many life-history traits such as preferring plant flowers and fruits. Furthermore, *H. armigera* and its descendant *H. zea* can reciprocally mate to produce viable offspring both in the lab (Hardwick, 1965; Laster and Sheng, 1995; Matthews, 1999) and in the field (Anderson et al., 2018). *H. armigera* can also mate with its old sister species *H. assulta* to produce normal males and females (*H. armigera* male × *H. assulta* female) or normal males and abnormal/sterile males (*H. armigera* female × *H. assulta* male) (Wang et al., 2004; Zhao et al., 2005; Tang et al., 2006). Nonetheless, their host ranges are dramatically different. The old sister species *H. assulta* is a specialist which feeds only on several Solanaceae plants including tobacco, tomato, eggplants and pepper, whereas the two descendants *H. armigera* and *H. zea* are generalists which feed on hundreds of plant species that belong to more than three plant families (Kogan et al., 1978; Fitt, 1989; Mitter et al., 1993; Zalucki et al., 1994; Jallow et al., 2004; Cho et al., 2008). Since the genome size of *H. zea* has been reported (Coates et al., 2016), we measured the genome sizes of *H. armigera* and *H. assulta* after comparing the suitability of six larval tissues for estimation of their genome sizes. We also compared the DNA ploidy (endoreplication) levels of host modification tissues [head (its mandibular gland) and salivary gland, two tissues secreting effectors into saliva to modify/repress plant defenses], host adaptation tissue (midgut, a digestive tissue containing detoxification enzymes to degrade toxic plant allelochemicals), and host use-independent tissues (hemolymph, male gonad and Malpighian tubules) of *H. armigera*. Our data show a trend of genome contraction along with expansion of host plant range in the group and higher levels of endoreplication in host modification tissues than in host adaptation tissues of *H. armigera*. These results suggest that *Helicoverpa* species appear to depend more on host modification than host adaptation to achieve polyphagy.

## Materials and Methods

### Insects

The laboratory colonies of *H. armigera* and *H. assulta* used in this study were simultaneously established with newly emerged *F*_0_ adults of each species developed from about 2,000 larvae of *H. armigera* (about 60%) or *H. assulta* (about 40%) (indistinguishable at larval stage) collected in tobacco fields in Xvchang (Henan, China) in June 2016 and had been reared for 8–10 generations on wheat germ-containing artificial diets (Waldbauer et al., 1984) in two separate growth chambers at 26 ± 0.5°C with a photoperiod of 16 h light: 8 h dark and a relative humidity of 75 ± 5% (for adults) or 50 ± 5% (for larvae). The wild type w1118 strain of *Drosophila melanogaster* was maintained on a corn culture medium under the same conditions.

### Isolation of Nuclei

We dissected and pooled the heads, hemolymphs, salivary glands, male gonads, midguts and Malpighian tubules from five last instar larvae of *H. armigera* to obtain one sample for each of the six tissues. We collected at least three samples for each of the six *H. armigera* tissues. For *H. assulta* and *D. melanogaster*, we prepared at least 3 samples of 5 larval (last instar larvae of *H. assulta*) or 12 adult (*D. melanogaster*) heads per sample.

We followed the standard protocol (Galbraith et al., 1983; Dolezel et al., 2007; Hanrahan and Johnston, 2011) to release and stain cell nuclei from each tissue sample. Briefly, we rinsed each sample in phosphate buffer saline and chopped it with a sharp blade (no chopping for hemolymph samples) in 50 μL Galbraith’s buffer [pH 7.0, containing 45 mM MgCl_2_, 20 mM MOPS, 30 mM sodium citrate, and 0.1% (v/v) Triton X-100] on ice. We then transferred the dissociated tissue into one 1.5 mL tube with 950 μL ice-cold Galbraith’s buffer containing 20 μg/mL RNase A and incubated on ice for 30 min with occasional shaking to release nuclei. Nuclei were separated from debris by filtering through a 40 μm nylon mesh and sedimented by centrifuge at 1,000 × *g* at 4°C for 5 min. The pelleted nuclei were resuspended in 500 μL ice-cold 50 μg/mL propidium iodide (PI) solution and held on ice in dark until analyzing them by flow cytometry.

### Flow Cytometry and Data Analysis

**FIGURE 1:**
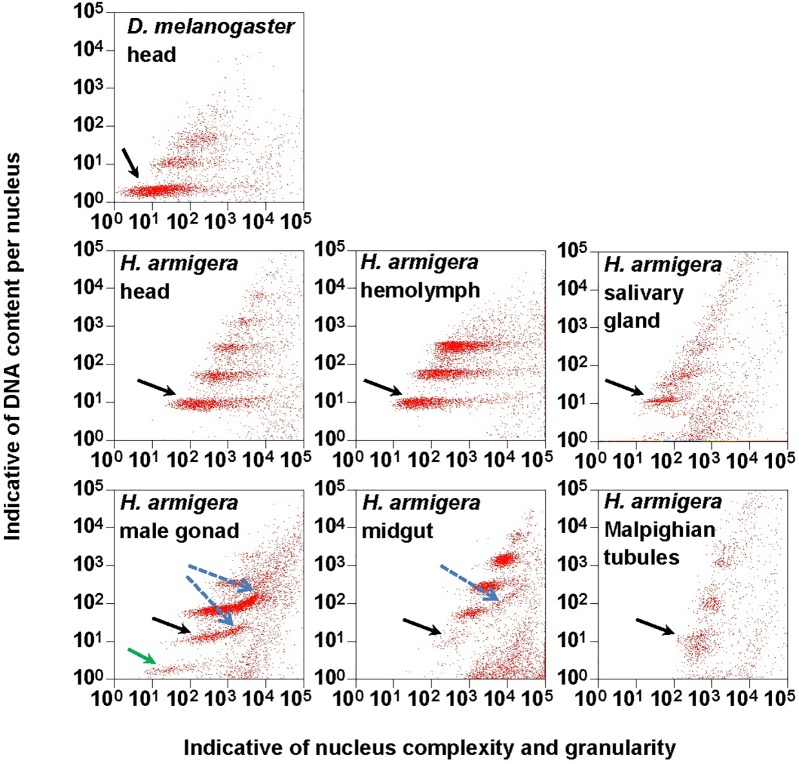
Clusters of endopolyploidy nuclei in the six larval tissues of *H. armigera*. DNA in nuclei were stained with the fluorescent dye propidium iodide (PI). PI-stained nuclei in each nucleus sample are clustered into different groups based on their side scatter signal (Indicative of shape complexity and granularity on *X*-axis) and PI fluorescence intensity (Indicative of DNA content per nucleus on *Y*-axis). A green arrow indicated a 1Cx nuclei cluster in male gonad. Black arrows indicated 2Cx nuclei clusters. Blue dashed arrows indicated right-hand tails.

Each PI-stained nucleus sample was analyzed on the MoFlo^TM^ XDP High Speed Cell Sorter and Analyzer (Beckman Coulter, CA, United States). The stained nuclei were excited by a 488 nm laser beam set to a power of 100 mW and the emitted PI fluorescence signals were captured at 630 nm in the detector Channel FL3. Summit Software (Beckman Coulter, CA, United States) was used to obtain the PI fluorescence histogram (Figure 3, 4) and the PI fluorescence vs. side scatter signal plot (Figure 1) of each nucleus sample. We used the fluorescence vs. side scatter signal plot to infer the DNA ploidy level of each sample. We used the mean fluorescence intensity of the 2Cx peak of each sample found in the corresponding PI fluorescence histogram (Figure 3, 4) to calculate the C-value (genome size) of each sample with the formula below:

$$\mathrm{Sample}\;1C\;\mathrm{value}\;(\mathrm{genome}\;\mathrm{size})=\frac{\mathrm{Mean}\;\mathrm{fluorescence}\;\mathrm{intensity}\;\mathrm{of}\;\mathrm{sample}\;2\mathrm{Cx}\;\mathrm{peak}}{\mathrm{Mean}\;\mathrm{fluorescence}\;\mathrm{intensity}\;\mathrm{of}\;\mathrm{reference}\;2\mathrm{Cx}\;\mathrm{peak}}\times \mathrm{reference}\;1C\;\mathrm{value},$$

where the reference C-value, i.e., the genome size of *D. melanogaster*, is 0.18 pg (Gregory and Johnston, 2008).

We used Chi-square test to compare the percentages of nuclei at different ploidy levels among various tissues. We used Student’s *t*-test to compare the C-values (genome sizes) of *H. armigera* and *H. assulta*, and to determine if the ratios of the average fluorescence intensity between two adjacent nuclei clusters are different from the expected 2.0 ratio. The C-values of the six larval tissues of *H. armigera* were analyzed by one-way ANOVA, followed by Fisher’s LSD test.

### Phylogenetic Analysis

The phylogenetic tree of the six heliothine group species (four *Helicoverpa* species and two *Heliothis* species) was generated based on their cytochrome oxidase subunit I (COI) gene sequences with MEGA 6.0 software using maximum likelihood method. The partial COI (1,486 bp) gene sequences of the six species were retrieved from GenBank (Accession No.: KF977797, KR149448, GU188273, KJ930516, NC_028539 and JN798950). The resultant tree agrees with their phylogenetic relationship reported previously (Mallet et al., 1993; Mitter et al., 1993; Fang et al., 1997; Behere et al., 2007; Cho et al., 2008; Pearce et al., 2017).

## Results

### Endopolyploidy Patterns of *H. armigera* Larval Tissues

The PI fluorescence (i.e., indicative of DNA content per nucleus on *Y*-axis) vs. side scatter signal (i.e., indicative of nucleus complexities and granularity on *X*-axis) plots (Figure 1) showed that both adult head of *D. melanogaster* (the reference sample) and six larval tissues (head, hemolymph, salivary gland, male gonad, midgut and Malpighian tubules) of *H. armigera* not only had a cluster of diploid nuclei (2Cx, indicated by black arrows in Figure 1), but also possessed at least two clusters of endopolyploid (4Cx and 8Cx) nuclei (Figure 1). Larval salivary gland exhibited a consecutive chain of endopolyploidy particles from 8Cx to higher than 32Cx, which is most likely resulted from breakage of extremely polyploid (Taylor et al., 1993). Larval head and midgut of *H. armigera* also had two tiny clusters of 16Cx and 32Cx endopolyploid nuclei, whereas male gonad of *H. armigera* also had a tiny cluster of haploid nuclei (1Cx, indicated by a green arrow in Figure 1).

**FIGURE 2:**
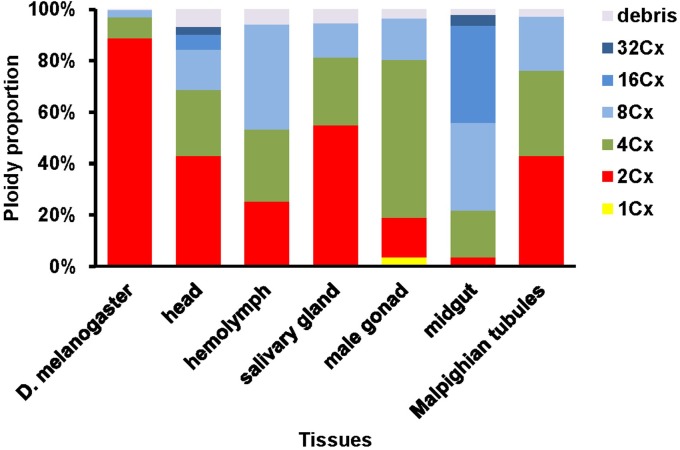
Nuclei composition of various ploidy classes in the six larval tissues of *H. armigera*.

We further quantified nuclei in each cluster of all the samples. Adult head of *D. melanogaster* contained 88.8, 8.3 and 2.8% of 2Cx, 4Cx and 8Cx nuclei, respectively (Figure 2). The nucleus composition of larval head, salivary gland, and Malpighian tubules of *H. armigera* followed a similar downward trend from 2Cx to 8Cx (salivary gland and Malpighian tubules) or 32Cx (larval head), but with significantly smaller proportion of 2Cx nuclei [43.2% in larval head (χ^2^ = 172, *p* < 0.0001), 54.9% in salivary gland (χ^2^ = 91.9, *p* < 0.0001) and 43.0% in Malpighian tubules (χ^2^ = 197, *p* < 0.0001)], relative to that of the adult head of *D. melanogaster* (Figure 2). On the contrary, the nucleus makeup of larval hemolymph of *H. armigera* exhibited an upward trend when the endopolyploid level increased from 2Cx to 8Cx. Unlike the two opposite patterns, the proportions of nuclei in larval male gonad and midgut of *H. armigera* increased with the rounds of endoreplication to the second highest endopolyploid level (61.4% at 4Cx for male gonad, 37.9% at 16Cx for midgut), but then dropped sharply at the highest endopolyploid level (16.2% at 8Cx for male gonad, 4.2% at 32Cx for midgut) (Figure 2).

We also calculated the ratios of the average fluorescence intensity between two consecutive ploidy levels of nuclei for all the samples. All of the ratios, except for the 2Cx/1Cx ratio for the larval male gonad of *H. armigera* (2.17 ± 0.069), were at least numerically less than the expected 2.0 ratio (Table 1), suggesting that under-replication is a widespread feature of polyploid *H. armigera* cells. The 4Cx/2Cx ratios of the six larval tissues of *H. armigera* and the adult head of *D. melanogaster* ranged from 1.82 of the larval salivary gland, a well-known under-replication tissue (Leach et al., 2000; Yarosh and Spradling, 2014), to 1.99 of the larval hemolymph. Only three (adult head, larval hemolymph, and larval male gonad) out of the seven 4Cx/2Cx ratios were not significantly less than the expected 2.0 ratio. In the larval salivary gland and Malpighian tubules of *H. armigera*, the ratios increased with the rounds of endoreplication, whereas in the other samples, the ratio decreased with the rounds of endoreplication (Table 1).

**Table 1:** The ratios of average fluorescence intensity between two adjacent clusters of nuclei in the adult head of *D. melanogaster* and the six larval tissues of *H. armigera*.

Species and tissue	*N*	2Cx/1Cx			4Cx/2Cx			8Cx/4Cx			16Cx/8Cx			32Cx/16Cx		
						
		Ratio	*SE*	*p*	Ratio	*SE*	*p*	Ratio	*SE*	*p*	Ratio	*SE*	*p*	Ratio	*SE*k	*p*
*D. melanogaster* head	3				1.94	0.019	0.088	1.66	0.050	0.021						
*H. armigera* head	3				1.94	0.015	0.048	1.93	0.012	0.026	1.85	0.012	0.006	1.79	0.005	0
*H. armigera* hemolymph	3				1.99	0.009	0.268	1.91	0.014	0.025						
*H. armigera* salivary gland	3				1.81	0.016	0.007	1.94	0.018	0.080						
*H. armigera* male gonad	3	2.17	0.069	0.127	1.89	0.038	0.104	1.78	0.043	0.037						
*H. armigera* midgut	3				1.95	0.005	0.008	1.84	0.013	0.007	1.85	0.023	0.022	1.71	0.006	0
*H. armigera* Malpighian tubules	3				1.82	0.031	0.029	1.93	0.058	0.329						


**N*, number of replicates; SE, standard error of ratio; *p, p* value of *t*-test.*

### Suitable Tissue for Estimation of the *Helicoverpa* Species Genome Size

**FIGURE 3:**
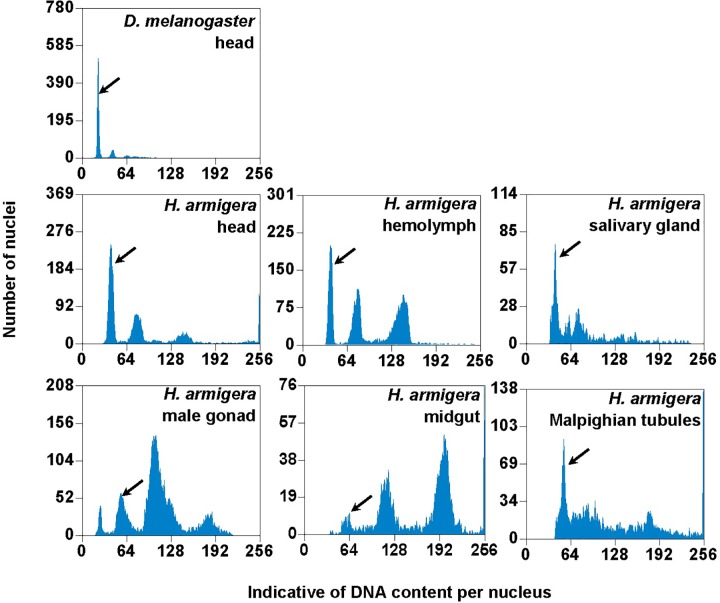
PI fluorescence histograms of the six larval tissues of *H. armigera*. DNA in nuclei were stained with the fluorescent dye propidium iodide (PI). The relative PI fluorescent intensities of all the PI-stained nuclei (Indicative of DNA content per nucleus on *X*-axis) in a given nucleus sample are plotted with the number of nuclei at given PI fluorescent intensity on *Y*-axis. Black arrows indicated 2Cx nucleus peaks.

We used the mean fluorescence intensity of the 2Cx peak of each sample found in the corresponding PI fluorescence histogram (Figure 3) to calculate the haploid DNA content, i.e., the C-value (genome size) of *H. armigera*. Overall, the C-values were significantly different among the six larval tissues of *H. armigera* (One-way ANOVA, *F*_5,12_ = 3.29, *p* = 0.0423) (Table 2). Fisher’s LSD test divided the six larval tissues into three different groups. The C-values estimated from the larval male gonad (0.465 pg), midgut (0.451 pg) and Malpighian tubules (0.469 pg) were not different from each other, but significantly larger than that of the larval hemolymph (0.381 pg). The C-values estimated from the larval head (0.402 pg) and salivary gland (0.415 pg) fell between the above two groups. Because the larval head exhibited an endopolyploidy pattern that was the most similar to that of the adult head of *D. melanogaster* (Figure 1–3), lacked obvious interference of impurities (Figure 1) and the 4Cx/2Cx over- or under-replication (Table 1), and had one of the lowest coefficient of variation (CV) (Table 2), we selected larval head to measure and compare the genome size of *H. armigera* and *H. assulta*.

**Table 2:** The C-values of the six larval tissues of *H. armigera*.

	Head	Hemolymph	Salivary gland	Male gonad	Midgut	Malpighian tubules
*N*	3	3	3	3	3	3
C-value (pg)	0.402^ab^	0.381^a^	0.415^abc^	0.465^c^	0.451^bc^	0.469^c^
SE (pg)	0.00187	0.00225	0.00129	0.0239	0.0149	0.0401
CV range (%)	3.69–6.44	3.23–6.17	6.22–8.87	7.5–10.2	6.31–7.68	4.93–9.20


*C-values with different letters are significantly different at *p* < 0.05 (One-way ANOVA, followed by Fisher’s LSD test). *N*, number of replicates; SE, standard error of C-value.*

### Genome Sizes of *H. armigera* and *H. assulta*

**FIGURE 4:**
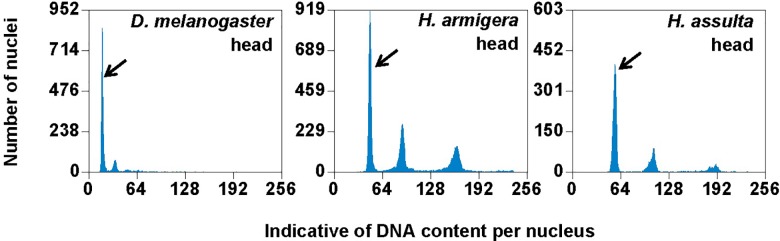
PI fluorescence histograms of larval heads of *H. armigera* and *H. assulta*. DNA in nuclei were stained with the fluorescent dye propidium iodide (PI). The relative PI fluorescent intensities of all the PI-stained nuclei (Indicative of DNA content per nucleus on *X*-axis) in a given nucleus sample are plotted with the number of nuclei at given PI fluorescent intensity on *Y*-axis. Black arrows indicated 2Cx nucleus peaks.

Flow cytometry analysis showed that the larval head of *H. assulta* and *H. armigera* shared a similar endopolyploidy pattern (Figure 4). The estimated C-values of *H. armigera* and *H. assulta* were 0.403 and 0.440 pg, respectively (Table 3). According to 1 pg = 978 Mb, *H. armigera* had a genome size of 394 Mb, which was significantly smaller than that (430 Mb) of *H. assulta* (*t*_9_ = -11.51, *p* < 0.0001).

**Table 3:** The estimated genome sizes of *H. armigera* and *H. assulta*.

	*H. armigera*	*H. assulta*
*N*	6	5
C-value (pg)	0.403^a^	0.440^b^
Genome size (Mb)	394^a^	430^b^
SE (Mb)	2.42	1.78
CV range (%)	3.69–6.73	2.69–5.68


*C-values and genome sizes with different letters are significantly different at *p* < 0.05 (Student’s *t*-test). *N*, number of replicates; SE, standard error of C-value.*

## Discussion

In order to verify if the positive correlation between genome size and host plant range revealed from the 21 distantly related lepidopteran herbivores by Calatayud et al. (2016) holds true among the three closely related *Helicoverpa* species, six larval tissues of *H. armigera* were used to determine a suitable tissue for estimation of their genome size by flow cytometry. Genome size of a given species estimated with different tissues may vary dramatically because of tissue differences in endopolyploidy pattern and level of over- or under-endoreplication (Trávníček et al., 2015), degree of difficulty to isolate intact nuclei (Dolezel et al., 2007), and endogenous compounds/debris capable of interefering with PI fluorescence staining of nuclei (Price et al., 2000; Loureiro et al., 2006; Dolezel et al., 2007; Greilhuber et al., 2007; Bennett et al., 2008; Greilhuber, 2008). Based on previous findings (Loureiro et al., 2006; Dolezel et al., 2007; Greilhuber et al., 2007), the occurrences of right-hand tails in the side scatter plots of larval male gonad and midgut (blue dashed arrows in Figure 1), and of right-skewed or shouldered 2Cx nucleus peaks in the PI fluorescence histogram of larval salivary gland, male gonad, midgut and Malpighian tubules (Figure 3) suggest that these four tissus contain fluorescence–strengthening debris. This is not only consistent with the larger genome sizes and CV values obtained from the four tissues (Table 2), but also agrees with the overestimation of the *Heliothis virescens* genome size using Malpighian tubules (Taylor et al., 1993). The occurance of left-skewed 4Cx and 8Cx nucleus peaks in the histogram plot of larval hemolymph (Figure 3) implies that it possesses fluorescence–weakening substances, consistent with the smallest genome size estimatd from this tissue (Table 2). Larval head, on the other hand, is a suitable tissue for estimation of the genome size of the two *Helicoverpa* species because it had the most similar endopolyploidy pattern to that of the adult head of *D. melanogaster* (Figure 1–3) and lacked obvious fluorescence-interfering debris (Figure 1) and 4Cx/2Cx over- or under-replication (Table 1). This result is in line with the recommendation of Hare and Johnston (2011).

**FIGURE 5:**
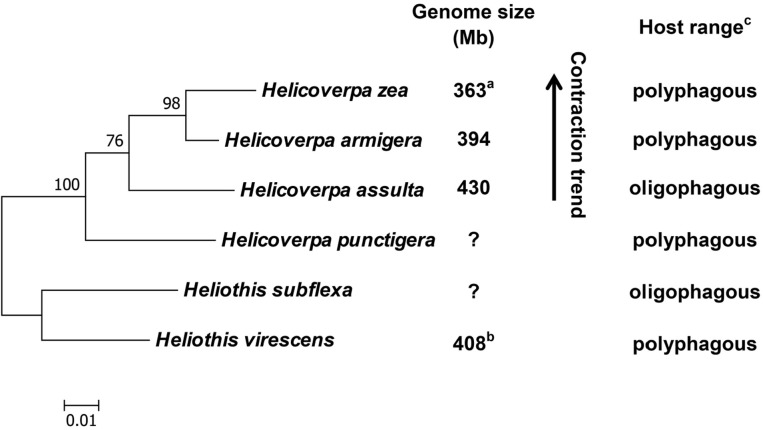
Phylogeny, genome size and host plant range of the *Helicoverpa* lineage. This maximum likelihood tree was constructed based on their cytochrome oxidase subunit I (COI) gene sequences in MEGA 6.0. and previous studies (Mallet et al., 1993; Mitter et al., 1993; Fang et al., 1997; Behere et al., 2007; Cho et al., 2008; Pearce et al., 2017). The partial COI (1,486 bp) gene sequences of the six species were retrieved from Genbank (Accession No.: KF977797, KR149448, GU188273, KJ930516, NC_028539 and JN798950). Numbers above the nodes indicate bootstrap values. The genome sizes of *H. zea* (a) and *H. virescens* (b) have been reported by Coates et al. (2016) and Taylor et al. (1993), respectively. The information about the host plant ranges of the six species (c) are obtained from Cho et al. (2008); Fitt (1989) and Mitter et al. (1993). Question marks: genome size unknown.

With previous estimation of the genome sizes of *H. zea* (363 Mb) (Coates et al., 2016) and *H. virescens* (408 Mb) (Taylor et al., 1993), we now have the genome size data for four out of the six heliothine group species included in the *Helicoverpa* phylogeny tree (Figure 5) based on their COI gene sequences and previous studies (Mallet et al., 1993; Mitter et al., 1993; Fang et al., 1997; Behere et al., 2007; Cho et al., 2008; Pearce et al., 2017). The *H. armigera* genome size we estimated by flow cytometry (394 Mb in Table 3) is ∼60 Mb larger than the recently published draft genome sequence of *H. armigera* (337 Mb) (Pearce et al., 2017). This discrepancy is likely to arise from interpopulation variation and/or sequencing gaps in heterochromatic regions. In any case, the evolution of the Solanaceae specialist *H. assulta* (430 Mb) from the generalist *H. virescens* (408 Mb) to the generalist *H. armigera* (394 or 337 Mb) and then to the generalist *H. zea* (363 Mb) (Kogan et al., 1978; Fitt, 1989; Mitter et al., 1993; Jallow et al., 2004; Cho et al., 2008) indicates that the expansion of host plant range is inversely correlated with the genome size in this closely related terminal lineage (Figure 5). This is contrary to the finding obtained from comparison of the 21 distantly related lepidopteran herbivores (Calatayud et al., 2016), but is consistent with the prediction of relying more on host modification than host adaptation to achieve host generalism (McNally et al., 2014).

Endopolyploidy has been implicated as a means of generating the extra copies of key fitness-enhancing genes to boost their RNA production in organisms with small genomes, particularly in tissues where those genes are expressed to perform their functions (Nagl, 1976; Bourdon et al., 2012; Scholes and Paige, 2014; Neiman et al., 2015). Caterpillar salivary glands and mandibular glands in the larval head are two major host plant modification tissues as they produce/secrete large amounts of saliva to modify host nutrients and repress host plant defenses when feeding (Rivera-Vega et al., 2017). Larval fat body is a host adaptation tissue as it produces various detoxification enzymes to cope with the diversity and unpredictability of plant defense allelochemicals (Li et al., 2002b). Larval midgut functions not only as a major host adaption tissue for digestion of plant tissues/nutrients and detoxification of plant defense allelochemicals, but also as a minor host modification tissue for contribution part of its luminal contents into saliva (Yoshinaga, 2016; Cheng et al., 2017). Our finding of 16Cx and 32Cx endopolyploidy nucleus clusters or the consecutive chain of endopolyploidy particles from 8Cx to higher than 32Cx in the larval head, midgut or salivary gland but not in the other three tissues (Figure 1, 2) and fat body (Taylor et al., 1993) supports that *H. armigera* rely more on host modification than on host adaptation although both are essential for expanding its host plant range. The fact that the *H. armigera*/*H. assulta* ratios of the total number of RNA-seq contigs are 1.88 for salivary gland, 1.20 for midgut and 1.15 for fat body (Wang et al., unpublished data) also implies that host modification probably plays a greater role than does host adaptation in the host plant expansion of *H. armigera*.

No matter whether the generalist *Helicoverpa* species rely more on host adaptation or host modification to expand host plant range, it is hard to believe that most of the unneeded host use-related genes had been lost from the genome when one population of the early diverged generalist *H. punctigera* evolved to become the Solanaceae plant specialist *H. assulta*, and then regained via gene duplication and subsequent neofunctionalization when one population of *H. assulta* evolved into the generalist *H. armigera* (Figure 5). Thus, we speculate that most of the host use-related genes have remained in the genome all the time, but the unneeded ones are pseudogenized or turned off due to increased TE proliferations (thus larger genome) in the specialist *H. assulta* and then reactivated due to deletions of TE insertions surrounding these genes (thus smaller genome) in the generalist *H. armigera*. Comparative analysis of *H. armigera* and *H. assulta* genomic sequences are required to test this speculation.

In conclusion, our data reveals that host plant range is negatively correlated with genome size in the closely related *Helicoverpa* lineage species. Consistent with this negative relationship, our results also imply that *Helicoverpa* species depend more on host modification than host adaptation to achieve polyphagy. However, it remains unclear whether the oscillations of host plant range in *Helicoverpa* species accompany with gain and loss or pseudogenization (off) and reactivation (on) of host use genes.

## Author Contributions

SZ and XL conceived and designed the experiments. SZ performed the experiments. XL and SZ analyzed the data. XL, SZ, XN, and SG wrote the manuscript. All authors have read and approved the manuscript for publication.

## Conflict of Interest Statement

The authors declare that the research was conducted in the absence of any commercial or financial relationships that could be construed as a potential conflict of interest.
